# Who Benefits from Government Healthcare Subsidies? An Assessment of the Equity of Healthcare Benefits Distribution in China

**DOI:** 10.1371/journal.pone.0119840

**Published:** 2015-03-17

**Authors:** Mingsheng Chen, Guixia Fang, Lidan Wang, Zhonghua Wang, Yuxin Zhao, Lei Si

**Affiliations:** 1 School of Health Policy & Management, Nanjing Medical University, Nanjing, China; 2 School of Health Administration, Anhui Medical University, Hefei, China; 3 National Health Development Research Center, Ministry of Health of the People’s Republic of China, Beijing, China; 4 Menzies Institute for Medical Research, University of Tasmania, Hobart, Australia; Monash University, AUSTRALIA

## Abstract

**Background:**

Improving the equitable distribution of government healthcare subsidies (GHS), particularly among low-income citizens, is a major goal of China’s healthcare sector reform in China.

**Objectives:**

This study investigates the distribution of GHS in China between socioeconomic populations at two different points in time, examines the comparative distribution of healthcare benefits before and after healthcare reforms in Northwest China, compares the parity of distribution between urban and rural areas, and explores factors that influence equitable GHS distribution.

**Methods:**

Benefit incidence analysis of GHS progressivity was performed, and concentration and Kakwani indices for outpatient, inpatient, and total healthcare were calculated. Two rounds of household surveys that used multistage stratified samples were conducted in 2003 (13,564 respondents) and 2008 (12,973 respondents). Data on socioeconomics, healthcare payments, and healthcare utilization were collected using household interviews.

**Results:**

High-income individuals generally reap larger benefits from GHS, as reflected by positive concentration indices, which indicates a regressive system. Concentration indices for inpatient care were 0.2199 (95% confidence interval [CI], 0.0829 to 0.3568) and 0.4445 (95% CI, 0.3000 to 0.5890) in 2002 (urban vs. rural, respectively), and 0.3925 (95% CI, 0.2528 to 0.5322) and 0.4084 (95% CI, 0.2977 to 0.5190) in 2007. Outpatient healthcare subsidies showed different distribution patterns in urban and rural areas following the redesign of rural healthcare insurance programs (urban vs. rural: 0.1433 [95% CI, 0.0263 to 0.2603] and 0.3662 [95% CI, 0.2703 to 0.4622] in 2002, respectively; 0.3063 [95% CI, 0.1657 to 0.4469] and −0.0273 [95% CI, −0.1702 to 0.1156] in 2007).

**Conclusions:**

Our study demonstrates an inequitable distribution of GHS in China from 2002 to 2007; however, the inequity was reduced, especially in rural outpatient services. Future healthcare reforms in China should not only focus on expanding the coverage, but also on improving the equity of distribution of healthcare benefits.

## Introduction

The equity of the distribution of government healthcare subsidies (GHS) within developing countries has recently received considerable attention [1–3]. Policymakers face ongoing challenges in this sector: if the distribution is equitable, they must demonstrate how GHS benefit the poor, and, if not, they must identify which factors affect distribution.

In 2003, a health care initiative was launched in China, and many more GHS have been appropriated to healthcare facilities [4]. However, it remains unclear whether GHS allocation has become more equitable since these policy revisions were implemented. Accordingly, the main objective of the current paper is to examine and compare the distribution equity of GHS before and after the country’s health care reform. China’s GHS comprise a budget appropriation to the healthcare sector that is intended to support the provision of widely accessible and relatively low-cost healthcare, especially to low-income persons. Due to rising healthcare costs and an out-of-pocket (OOP) financing mechanism, it is increasingly difficult for poor patients, especially those who are in greater need of care, to pay privately for healthcare in China. Affordable prices for healthcare services such as ambulatory care, hospitalization, and medicines have been set by the Chinese authorities in order to make healthcare financially accessible. At the same time, the state provides healthcare facilities with funds to offset the deficits created by the difference between low healthcare prices and high hospital costs. In other words, Chinese patients stand to benefit from GHS as long as they consume healthcare services in public hospitals [5, 6]. Low-income and disadvantaged groups should benefit the most from these GHS initiatives, but it is often upper-income patients who reap the largest benefits from public spending programs [7–9]. In addition, the provision of public benefits to the entire population requires that policymakers and researchers investigate and analyze how healthcare sector resources are targeted, delivered, and distributed in China; it is particularly important to determine how well these subsidies and services benefit the intended recipients (i.e., people of lower socioeconomic status).

A systematic review of GHS provision in several countries outside of China revealed clear benefits for wealthier citizens [10], a finding that some investigators have attributed to budgetary allocations within tertiary healthcare services and to a lower overall utilization of healthcare services by poorer populations [11, 12].

Since the initiation of market-oriented economic reforms in the early 1980s, China has experienced an unprecedented transformation of its social and economic institutions. However, a smooth transition to a market-oriented economy requires the structural modification of all state-level functions—including budget resource allocations that limit state participation in the healthcare sector. Government expenditure on healthcare has rapidly declined following the decentralization of financial responsibility for managing healthcare [13, 14]. In the early 1980s, government funding accounted for about 60% of public hospitals’ revenues, but this had shrunk to 24.73% by 2008 [15]. To compensate for the effects of decreased public appropriations and to encourage financial incentives from healthcare institutions, public hospitals were allowed to determine independently the prices of most healthcare services and medicines. Revenue thus generated by hospitals was intended to address operating deficits and operational costs. However, these policies promoted aggressive drugs sales and the administration of unnecessary treatments, which led to increases in patients’ healthcare expenditures. At the same time, the healthcare financing mechanisms shifted primarily toward OOP payments because the government had reduced GHS [16, 17]. Changes in the government budget and funding of healthcare thus influenced access to medical services and GHS distribution among different populations. In addition, preferential coverage and high reimbursement rates by public health insurance for rich citizens in both urban and rural areas, alongside low coverage rates for the poor populations, resulted in higher healthcare utilization among those who were economically better off. As described above, changes in GHS distribution created dissimilar opportunities to obtain healthcare benefits for different socioeconomic groups.

Confronted with this situation, growing efforts are being made to improve GHS utilization by lower-income groups—especially since the publication of the *World Health Report 2000*, which stated that China had the most inequitable healthcare system in the world [18]. In 2003, China implemented new healthcare-specific policies and launched several healthcare sector reforms, including increased government healthcare inputs, an expansion of public health insurance coverage, and lower user fees. For example, the 2003 New Rural Cooperative Medical Scheme (NRCMS) was established to rebuild health insurance and overhaul healthcare provision in rural areas following the dissolution of the earlier Rural Cooperative Medical Scheme (CMS) in the late 1980s. Since its formation, China’s authorities have sanctioned additional public spending on the NRCMS, which has achieved high rural coverage, with the proportion of this population insured increasing from 9.64% in 2002 to 94.44% in 2007 (Table 1). At the same time, urban health insurance was also renovated. The existing basic insurance program—Urban Worker’s Basic Medical Insurance (UWBMI)was implemented in all conurbations, covering 30.43% and 61.04% of workers in 2001 and 2007, respectively [19]. In addition, in 2007, an Urban Resident’s Basic Medical Insurance (URBMI) was established to provide coverage for the unemployed, children, students, and elderly persons without pensions [20]. Both insurance programs were funded by individuals, with subsidies granted by the government as required [21]. However, it remains difficult to determine whether GHS distribution benefits the poor, especially given the current lack of empirical evidence regarding the actual degree of inequality in healthcare utilization. Meanwhile, inherent flaws in the revised approach to GHS have been identified, representing another challenge to policymakers as these practices currently exemplify the country’s “hoped-for healthcare reform”.

**Table 1:** Descriptive statistics and socioeconomic characteristics by income quintile (2002, 2007).

Year	Income quintiles	No. of surveyed households		No. of surveyed individuals		Per capita expenditures ^a^ ^,^ ^b^		Insurance coverage (%)		Reported illness (%)		Sought medical care (%)		Outpatient OOP ^a^ ^,^ ^b^		Inpatient OOP ^a^ ^,^ ^b^	
		Urban	Rural	Urban	Rural	Urban	Rural	Urban	**Rural**	**Urban**	**Rural**	**Urban**	**Rural**	**Urban**	**Rural**	**Urban**	**rural**
**2002**	Q_1_	394	394	1172	1538	4225.15	2094.54	4.95	7.10	11.09	10.60	85.71	58.33	271.64	110.46	3195.40	657.51
	Q_2_	395	395	1172	1544	7489.53	3905.50	14.71	8.64	11.86	11.08	79.07	68.14	302.22	179.34	5921.86	1086.26
	Q_3_	396	395	1173	1545	10523.18	5250.52	23.72	6.95	9.89	9.64	87.65	79.84	280.11	193.17	2728.79	911.09
	Q_4_	394	394	1172	1520	14264.82	6742.20	36.04	11.91	9.56	11.05	89.87	85.82	280.65	253.16	3243.69	1403.42
	Q_5_	395	394	1172	1556	23903.86	12861.29	55.97	13.62	12.37	13.11	92.08	88.83	409.65	568.71	2712.58	3914.78
	Total	1974	1972	5861	7703	12081.04	6182.51	27.09	9.64	10.95	11.10	87.03	77.70	333.62	308.33	3410.14	1904.21
**2007**	Q_1_	395	395	1116	1480	7855.63	3693.19	70.79	95.41	7.62	7.77	86.21	71.43	161.50	87.00	2858.15	575.12
	Q_2_	397	397	1119	1469	12126.31	5862.79	68.90	91.76	7.69	9.80	88.52	84.00	155.27	135.40	4245.40	1728.38
	Q_3_	396	395	1112	1488	16116.38	7373.86	71.58	93.75	7.37	9.95	88.33	77.31	176.10	249.57	3211.63	1203.56
	Q_4_	396	398	1119	1475	21288.24	10203.53	77.30	95.12	10.28	13.02	93.83	81.17	212.48	276.63	3213.00	1316.04
	Q_5_	395	394	1115	1480	33958.12	16746.58	82.33	96.15	12.91	11.35	85.86	83.22	442.09	359.15	6657.09	2847.16
	Total	1979	1979	5581	7392	18265.99	8777.84	74.18	94.44	9.17	10.38	88.58	79.97	224.18	227.62	4522.43	1819.13

Data source: Author’s calculations from household surveys.

^a^ Expenditures are presented in Chinese yuan (CNY).

^b^ 2002 nominal prices adjusted to real prices in 2007 according to China’s Consumer Price Index (CPI).

Variations in the extent of China’s GHS equity over the years have not been reviewed, but such research would shed light on the positive and negative effects of GHS utilization following the implementation of the country’s healthcare reform measures. These issues raise further questions for both policymakers and researchers, such as: How can equity in healthcare reform be assessed? Has equitable distribution been achieved? Who has benefitted from GHS following the implementation of recent policy reforms? Addressing these concerns requires the implementation of financial risk protection strategies to ensure that the poor and other vulnerable groups benefit from GHS, thereby reducing inequity in healthcare services utilization.

## Materials and Methods

### Ethics statement

This study was approved by the Academic Research Ethics Committee of Nanjing Medical University. Because more than 25,000 individuals were interviewed, including illiterate and older persons, verbal informed consent was obtained during household interviews. A list of the names of all the selected interviewees in a single community was printed on a piece of paper. Before the interview, the respondents provided verbal informed consent after the trained data collectors clearly read the informed consent form and explained the study objectives. With the permission of the respondents, the data collectors checked the box next to their names in the presence of the respondents and supervisor. Consequently, the Academic Research Ethics Committee waived the need for written informed consent from the participants.

### Data sources

Our data were obtained using two rounds of national household surveys completed in Gansu province, Northwest China. These surveys were conducted in 2003 and 2008 to collect information from the years 2002 and 2007. Gansu is an impoverished province with a population of more than 26 million people [22]. A total of 15 of the province’s cities and counties were selected using multistage stratified random sampling; then, 8 communities were selected from each city or county, according to economic status and geographic distribution. In each community, 33 households were randomly selected and members of the selected families were interviewed by trained data collectors. The incapacitated and children who were too young to be interviewed on their own were interviewed through their guardians. In 2003, 1,974 households with 5,861 individuals in urban areas and 1,972 households with 7,703 individuals in rural areas were enrolled and surveyed. In 2007, data from 1,979 households with 5,581 individuals in urban areas and 1,979 households with 7,392 individuals in rural areas were effectively collected using the survey. The descriptive and socioeconomic characteristics of each income quintile are summarized in Table 1.

The study’s surveys included a series of socioeconomic and demographic questions, covering household expenditure; urban–rural classification; household goods; and number, sex, age, education, and employment status of household members. Per capita household expenditures were adjusted to adult equivalence and used as a measure of living standard [23]. The data used to assess per capita GHS utilization were obtained from two sources: (1) the household surveys, which recorded information about healthcare utilization including outpatient visits, length of hospital stays (inpatient days were reported for each 12-month recall period and outpatient visits for the preceding 2 weeks), and healthcare facility levels (e.g., municipal, county, or township hospital; traditional Chinese medicine hospital; community health center); and (2) administrative and survey data from each local healthcare facility level, developed from their annual financial reports, on outpatient care visits, yearly income, and inpatient days(Table 2).

**Table 2:** Administrative and survey data on hospital income and healthcare utilization in Gansu province (2002, 2007).

Year	Faculty	Outpatient income (yuan)		Inpatient income (yuan)		Outpatient visit (person-time)		Inpatient day	
		Administrative data	Survey data	Administrative data	**Survey data**	**Administrative data**	**Survey data**	**Administrative data**	**Survey data**
**2002**	General hospital at municipal level	12804.67 × 104	375410.00	33626.3 × 104	946758.00	313.04 × 104	5421.00	211.23 × 104	4103.00
	General hospital at county level	10481.69 × 104	307938.00	18645.32 × 104	535964.00	670.03 × 104	10903.00	248.98 × 104	4936.00
	Urban primary health center	298.04 × 104	8747.00	123.01 × 104	3663.00	19.39 × 104	315.00	4.18 × 104	76.00
	Rural primary health center	4764.52 × 104	128587.00	2413.37 × 104	61949.00	1069.84 × 104	18126.00	70.39 × 104	1408.00
	Traditional Chinese medicine hospital at municipal level	1108.20 × 104	33380.50	2906.63 × 104	79836.00	47.00 × 104	793.00	29.03 × 104	463.00
	Traditional Chinese medicine hospital at county level	2680.22 × 104	71628.00	3107.45 × 104	89491.00	61.52 × 104	1265.00	47.78 × 104	1018.00
**2007**	General hospital at municipal level	41928.22 × 104	646118.00	106152.40 × 104	1679015.00	566.24.00 × 104	4508.00	412.16 × 104	3235.00
	General hospital at county level	18942.40 × 104	311904.00	37228.23 × 104	577839.00	550.81 × 104	4085.00	278.92 × 104	2289.00
	Urban primary health center	539.87 × 104	8919.00	387.45 × 104	6928.00	63.89 × 104	468.00	3.75 × 104	26.00
	Rural primary health center	8629.39 × 104	142979.00	8889.26 × 104	133601.00	1125.19 × 104	8357.00	174.58 × 104	1430.00
	Traditional Chinese medicine hospital at municipal level	299.76 × 104	4119.00	10105.36 × 104	169836.00	113.50 × 104	993.00	60.09 × 104	451.00
	Traditional Chinese medicine hospital at county level	255.62 × 104	4539.50	8632.58 × 104	163541.00	206.48 × 104	1843.00	81.49 × 104	669.00

### Statistical analysis

#### Calculation of Unit Subsidy

Although the GHS data were collected at the healthcare facility level, it was not possible to obtain separate data on GHS for outpatient and inpatient services. As mentioned above, patients receive government subsidies when they consume healthcare in Chinese hospitals, and hospitals provide both outpatient and inpatient care in China. In other words, patients receive outpatient and inpatient subsidies from the government when they consume outpatient and inpatient care, respectively. Because outpatient and inpatient care were both provided in hospitals, we inferred that the proportion of subsidies between outpatient and inpatient services could be estimated using the ratio of outpatient and inpatient income. This method is consistent with Huang et al. and Zhao et al., who researched the estimation of outpatient and inpatient subsidies on Chinese hospitals [5, 6]. Then, the unit subsidy at each healthcare facility level was calculated by dividing total service-specific subsidies by the total outpatient visits or inpatient days. The subsidy for each individual was total healthcare utilization multiplied by the unit subsidy at each facility level.

The unit subsidy at each healthcare facility level was calculated as follows [24]: *c*_*kj*_ = *s*_*kj*_ / *n*_*kj*_ where *s*_*kj*_ indicates the total subsidies for service *k* at healthcare facility level *j*, *n*_*kj*_ represents the total unit of health service use, measured by outpatient visits or inpatient days, and *c*_*kj*_ represents the unit subsidy when individual *i* receives service *k* at healthcare facility level *j*.

The service-specific subsidy received by an individual was determined as follows:

$$s_{ki}=\sum_{j}q_{kji}c_{kj}$$

where *q*_*kji*_ indicates the quantity of service *k* utilized by individual *i* at healthcare facility level *j*. The total subsidy received by an individual was calculated as follows:

$$s_{i}=\sum_{k}a_{k}q_{kji}c_{kj}$$

where *a*_*k*_ are scaling factors that standardize utilization recall periods across services. For example, one might standardize on the recall period that applies for the service accounting for the greatest share of the subsidy; where this is inpatient care, reported over a 1-year period, then *a*_*k*_ = 1 for inpatient care and *a*_*k*_ = 26 for outpatient services reported over a 2-week period.

#### Benefit Incidence Analysis

There are several methods that allow researchers to track whether public spending benefits the poor, including benefit incidence analysis (BIA). Developed by the World Bank in the 1970s, BIA uses a progressivity index to measure the extent of equity. It provides an important perspective for assessing the distributional effects of public spending by combining data on household use and hospital-related costs [25, 26], and it can evaluate GHS distribution among different groups in the population, especially those with different income levels. The main objective of the current study was to examine and compare the distribution equity of GHS before and after China’s healthcare reform, and BIA can be used to determine whether public spending is progressive or if low-income groups receive a larger share of the benefits from government spending than high-income groups [27, 28].

**Fig 1:**
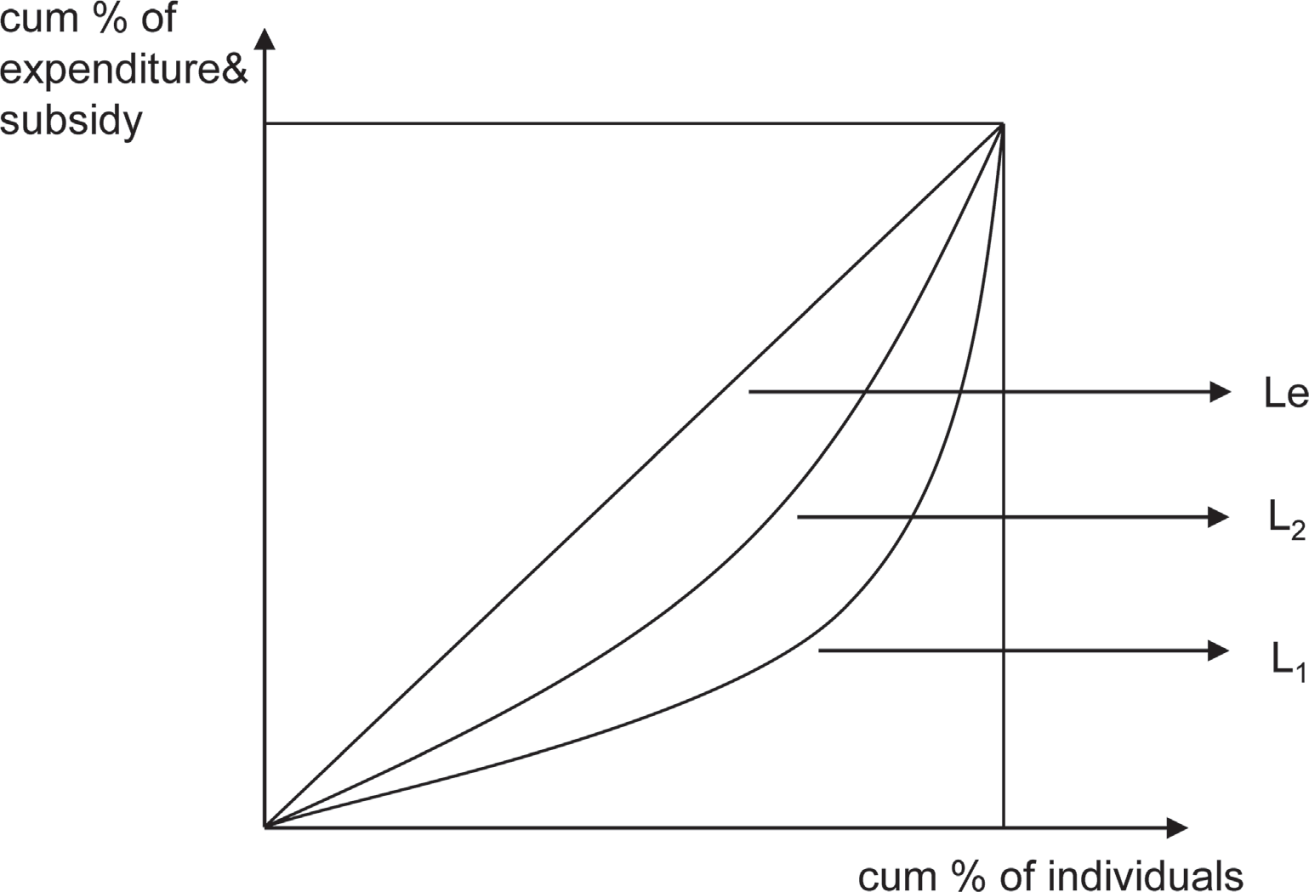
Conceptual cumulative concentration curve for government subsidies in terms of healthcare and income. Conceptual cumulative concentration curve for government subsidies in terms of healthcare and per capita income is shown. The concentration curve plots the cumulative percentage of health subsidy (y-axis) against the cumulative percentage of the population (x-axis). Population is ranked according to living standard, from the poorest to the richest. The concentration index is measured as twice the area between the concentration curve, L_1_, and the line of equality, L_e_ (the 45° line running from the bottom-left corner to the top-right). The Lorenz curve (L_2_) represents the relationship between the cumulative percentage of per capita income and the cumulative percentage of the population, which is measured by the Gini coefficient.

Fig. 1 shows the conceptual cumulative concentration curve for GHS across individuals and living standards. This concentration curve plots the cumulative percentage of health subsidies (y-axis) versus population (x-axis), thereby ranking living standards from those of the poorest to the richest individuals. The concentration index (*C*) is measured as twice the area between the concentration curve, *L*_*1*_, and the equality line (*L*_*e*_; the 45° line running from the bottom left corner to the top right).

Objectives should be properly set and target distribution must be referenced to appropriately evaluate the distribution of GHS. One alternative is to compare the distribution of subsidies to population shares to estimate the absolute progressivity of government spending [29]; that is, whether everyone, regardless of their living standard, receives exactly the same share of GHS or not. This is equal to comparing *L*_*1*_ and *L*_*e*_. If *L*_*1*_ lies above (below) *L*_*e*_, the distribution is pro poor (pro rich); thus, the subsidies narrow (widen) the absolute rich–poor welfare gap, which indicates strong progressivity (strong regressivity). A negative *C* value indicates a disproportionate concentration of GHS among the poor, and *L*_*1*_ accordingly lies above *L*_*e*_. If *L*_*1*_ lies further above *L*_*e*_, it indicates that the subsidy is even more concentrated among the poor, and *C* will be even larger (and vice versa).

However, using *C* is only appropriate if the distributional goal is to close the absolute rich–poor welfare gap. If the objective is to close the relative gap, it indicates that the subsidy reduces inequality (weak progressivity), and the share of subsidies received by the poor should exceed their share of the living standard. That is, *L*_*1*_ lies above the Lorenz curve (*L*_*2*_) [30].

L_2_ represents the relationship between the cumulative percentage of living standards and that of the population, which is measured by the Gini coefficient (*G*) of living standards and defined as twice the area between *L*_*2*_ and *L*_*e*_. Moreover, the Kakwani index (π_*k*_), which evaluates the relative gap in poor-rich subsidies, is defined as twice the area between *L*_*2*_ and *L*_*1*_:

$$\begin{array}{l} \pi_{k}=2\int_{0}^{1}\left[L_{2}-L_{1}\right]dp\\ \pi_{k}=2\int_{0}^{1}\left[L_{e}-L_{1}\right]dp-2\int_{0}^{1}\left[L_{e}-L_{2}\right]dp\\ \pi_{k}=C-G \end{array}$$

That is, *π*_*k*_ is the difference between *C* and *G*, and indicates the degree of GHS progressivity. A positive *π*_*k*_ value, where L_1_ lies below L_2_, indicates progressive distribution in which the poor receive a smaller proportion of benefits than the proportion of socioeconomic values they possess, and vice versa. For simplification, *π*_*k*_ >0 and *π*_*k*_ <0 indicate that GHS is regressive or progressive, respectively.

In addition, dominance testing was incorporated into BIA. To determine if GHS reduced inequity—whether low-income individuals received a larger share of subsidies than the wealthy relative to their living standards—a test was conducted to determine if *L*_*1*_ dominates (i.e., lies above) L_2_. For the dominance tests, the standard errors and differences of the curve ordinates were computed, allowing for dependence between curves when appropriate [24, 31]. Multiple comparisons were performed, with null defined as indistinguishable curves [32, 33].

## Results

Table 3 shows the quintile-based shares of subsidies spent on outpatient and inpatient care and total subsidies across the entire population in two separate years (2002 and 2007) and two regions (urban and rural areas). Three results (distribution of benefits, *C* and *π*_*k*_ values with 95% confidence intervals [CIs], and concentration curves) describe the benefits of government subsidies. Table 3 also shows differences in the Kakwani index values in different regions and times.

**Table 3:** Distribution of government healthcare subsidies by income quintile, Gini/concentration index (CI), and Kakwani index.

Year	Area	Income quintiles	Per capita income	**Outpatient care**	**Inpatient care**	**Total subsidy**
**2002**	**Urban (A)**	Q1 (poorest)	6.57%	15.32%	9.79%	11.94%
		Q2	11.16%	19.81%	24.35%	22.58%
		Q3	16.09%	17.07%	16.93%	16.98%
		Q4	23.83%	22.26%	15.11%	17.90%
		Q5 (richest)	42.35%	25.55%	33.82%	30.60%
		Gini/CI	0.4312**	0.1433	0.2199	0.1900
		*SE* ^†^	(0.0032)	(0.0597)	(0.0699)	(0.0520)
		95% CI‡	(0.4249, 0.4375)	(0.0263, 0.2603)	(0.0829, 0.3568)	(0.0881, 0.2920)
		Kakwani	-	-0.2879	-0.2113	-0.2412
		*SE*		(0.0598)	(0.0699)	(0.0521)
		95% CI		(-0.4051, -0.1707)	(-0.3484, -0.0743)	(-0.3433, -0.1391)
		Dominance test				
		—against 45° line		None	D-	D-
		—against Lorenz curve		D+	None	D+
	**Rural(B)**	Q1 (poorest)	6.48%	7.95%	7.12%	7.51%
		Q2	12.09%	11.14%	15.19%	13.28%
		Q3	16.30%	19.23%	13.68%	16.29%
		Q4	20.94%	23.31%	17.83%	20.42%
		Q5 (richest)	44.20%	38.37%	46.17%	42.50%
		Gini/CI	0.4443**	0.3662*	0.4445	0.2350*
		*SE*	(0.0155)	(0.0490)	(0.0737)	(0.0390)
		95% CI	(0.4140, 0.4747)	(0.2703, 0.4622)	(0.3000, 0.5890)	(0.1586, 0.3113)
		Kakwani	-	-0.0781	0.0001	-0.2094*
		*SE*	-	(0.0512)	(0.0749)	(0.0415)
		95% CI		(-0.1785, 0.0223)	(-0.1466, 0.1469)	(-0.2907, -0.1280)
		Dominance test				
		—against 45° line		D-	D-	D-
		—against Lorenz curve		None	None	None
**2007**	**Urban(C)**	Q1 (poorest)	7.68%	8.60%	5.53%	6.07%
		Q2	12.17%	16.23%	16.29%	16.28%
		Q3	16.75%	15.82%	16.23%	16.16%
		Q4	23.62%	26.75%	23.96%	24.45%
		Q5 (richest)	39.78%	32.60%	37.99%	37.04%
		Gini/CI	0.3880**	0.3063	0.3925	0.3773
		*SE*	(0.0032)	(0.0717)	(0.0713)	(0.0620)
		95% CI	(0.3818, 0.3943)	(0.1657, 0.4469)	(0.2528, 0.5322)	(0.2558, 0.4988)
		Kakwani	-	-0.0818	0.0045	-0.0107
		*SE*	-	(0.0716)	(0.0711)	(0.0618)
		95% CI		(-0.2221, 0.0586)	(-0.1350, 0.1439)	(-0.1319, 0.1104)
		Dominance test				
		—against 45° line		D-	D-	D-
		—against Lorenz curve		None	None	None
	**Rural(D)**	Q1 (poorest)	7.72%	18.57%	5.09%	8.44%
		Q2	12.40%	24.58%	14.42%	16.94%
		Q3	16.75%	21.29%	13.67%	15.56%
		Q4	22.54%	15.91%	27.63%	24.72%
		Q5 (richest)	40.58%	19.65%	39.20%	34.34%
		Gini/CI	0.3910**	-0.0273	0.4084	0.3002*
		*SE*	(0.0035)	(0.0729)	(0.0564)	(0.0471)
		95% CI	(0.3842, 0.3979)	(-0.1702, 0.1156)	(0.2977, 0.5190)	(0.2079, 0.3925)
		Kakwani	-	-0.4184	0.0173	-0.0908*
		*SE*	-	(0.0730)	(0.0564)	(0.0471)
		95% CI		(-0.5615, -0.2753)	(-0.0932, 0.1279)	(-0.1831, 0.0015)
		Dominance test				
		—against 45° line		None	D-	D-
		—against Lorenz curve		D+	None	None
**Inequalitydifference**	**Δ(urban**–**rural)**	2002 (A–B)	-	-0.2098	-0.2115	-0.0318
		Dominance test		None	None	None
		2007 (C–D)	-	0.3366	-0.0129	0.0801
		Dominance test		None	D-	None
	**Δ(2007–2002)**	Urban (C–A)	-	0.2061	0.2158	0.2304
		Dominance test		None	None	None
		Rural (D–B)	-	-0.3403	0.0172	0.1186
		Dominance test		D+	None	None

Notes: “None” indicates failure to reject the null hypothesis that curves are indistinguishable at the 5% significance level. “D+”/“D−”indicates that the concentration curve dominates (is dominated by) the Lorenz curve or concentration curve in one year or area and dominates (is dominated by) the other in another year or area.

* p < 0.05

** p < 0.01

^†^ standard error

^‡^ 95% confidence interval.

In both 2002 and 2007, all *C* values were positive except for rural outpatient care in 2007, suggesting that a greater proportion of the subsidies for both outpatient and inpatient services was generally allocated to the rich rather than to the poor. In the same region in the same year, *C* for ambulatory care was smaller compared with that for in-hospital services. This result indicates that, compared with outpatient care, the distribution of hospital-based care among all regions demonstrated an obvious pro-rich bias.

Between 2002 and 2007, the rich–poor economic gap narrowed in both urban and rural areas due to decreasing *G*. While economic development was similar in both regions during this period, pro-rich GHS trends moved in opposite directions in urban and rural areas. In cities, *C* increased from 0.1433 (95% CI = 0.0263 to 0.2603) to 0.3063 (95% CI = 0.1657 to 0.4469) for outpatient care, and from 0.2199 (95% CI = 0.0829 to 0.3568) to 0.3925 (95% CI = 0.2528 to 0.5322) between 2002 and 2007. On the other hand, *C* in rural areas for outpatient care in the same period decreased from 0.3662 (95% CI = 0.2703 to 0.4622) to −0.0273 (95% CI = −0.1702 to 0.1156). In conclusion, not only did *C* significantly decrease, the bias of the subsidies became increasingly pro poor.

Although *C* is a summary measure of absolute subsidy progressivity, closing the absolute rich–poor gap (i.e., allocation of a larger share of subsidies to the poor in absolute terms) is challenging because the poor receive far fewer medical services than do the rich. The Kakwani index can be used as a summary measure of weak progressivity; to determine if GHS reduces inequality (i.e., weak progressivity), *π*_*k*_ was used to estimate subsidy equity.

**Fig 2:**
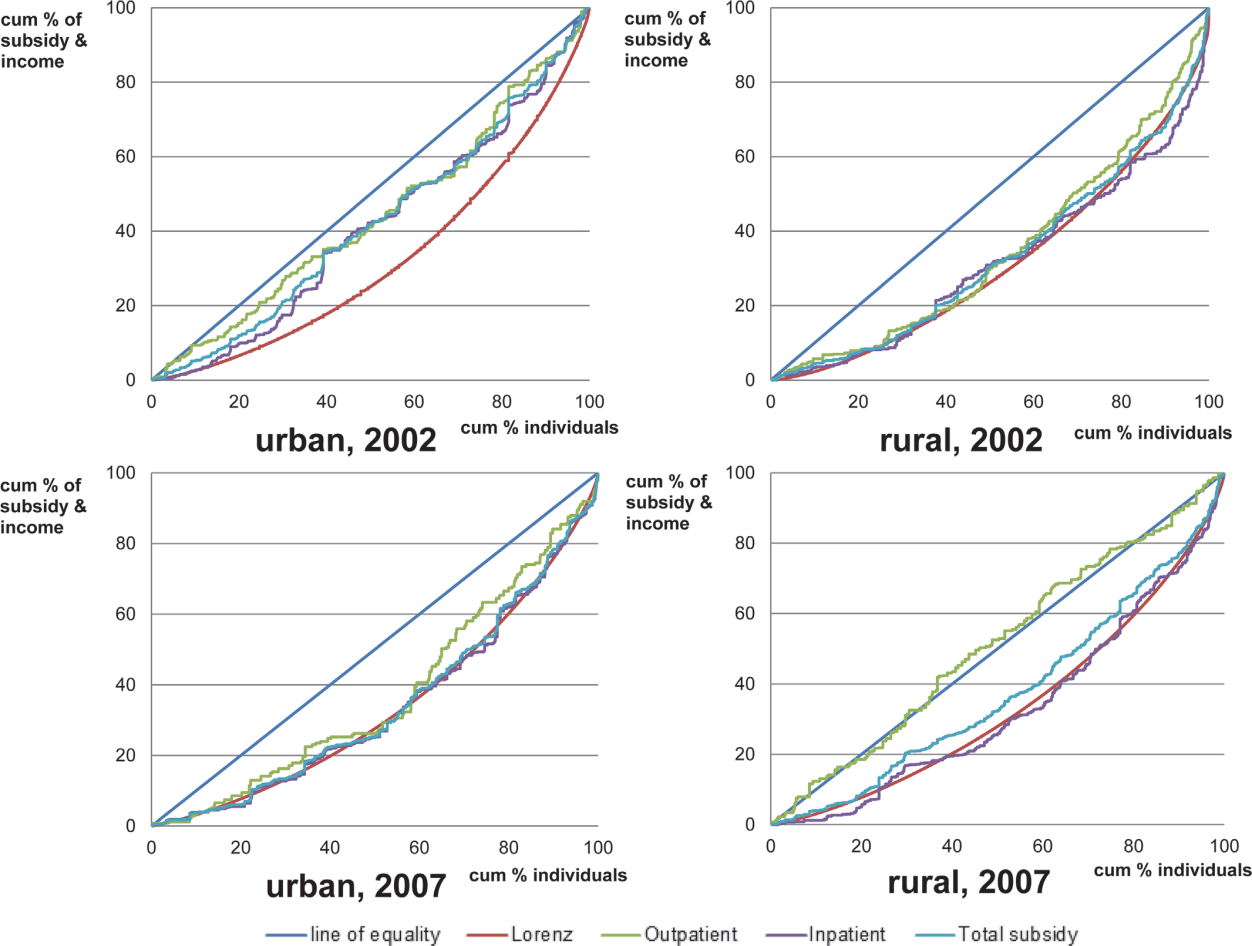
Concentration curve for government subsidies in terms of health care and income. Actual cumulative concentration curve for government outpatient, inpatient, and total healthcare subsidies is shown. Lorenz curves for 2002 and 2007 data in both urban and rural areas are shown.

In all cases of ambulatory care, *π*_*k*_ values remained negative over the study period, indicating that subsidies to outpatients were progressively distributed by socioeconomic status. However, except for urban healthcare facilities in 2002, the *π*_*k*_ values for inpatient care were generally positive. The results of our analysis indicate that inpatient GHS not only failed to close the absolute gap, it also did not bridge the relative rich–poor gap. The Lorenz income and concentration curves (Fig. 2) illustrate GHS progressivity.

Table 3 shows the improvements and setbacks in GHS equity from 2002 to 2007 based on differences in *π*_*k*_ values by region and time. First, comparison of urban–rural differences shows that, in 2002 (rows A–B), the differences in subsidies to outpatients (−0.2098) and inpatients (−0.2115) were both negative; in 2007 (rows C–D), the differences in subsidies for outpatients were positive (0.3366) and were slightly negative (−0.0129) for inpatients. These results imply that subsidies to urban healthcare facilities in 2002 were more effective in reducing inequality than subsidies to rural facilities, but that the difference became positive (outpatient care) or slightly negative (inpatient care) by 2007. This finding demonstrates that inequality-reducing performance in rural areas was not worse than in urban areas, especially for outpatient care. The differences between 2002 and 2007 also show that, in urban areas (rows C–A), differences in subsidies for outpatients (0.2061) and inpatients (0.2158) were both positive, indicating that the inequality-reducing performance of GHS had regressed. The differences in outpatient and inpatient rural healthcare facilities (rows D–B) were −0.3403 and 0.0172, respectively. The outpatient care subsidies in rural healthcare facilities changed significantly between 2002 and 2007, with inequality-reducing effects. Dominance tests resulted in concentration curves for rural outpatient care in 2007 and 2002, indicating that the poor received more GHS than the rich over the same period.

## Discussion

Do China’s GHS benefit the poor? The short answer is “not yet”. Under the strict standard of strong progressivity, in which the distribution of subsidies within China is determined in terms of population shares, almost all *C* values in both urban and rural areas were positive in 2002 and 2007, indicating that GHS are concentrated among the rich. Under the less demanding standard of weak progressivity, in which the distribution of subsidies is compared in terms of the share of welfare, inequality-reducing effects were found in many types of health services. Nevertheless, such effects diminished over the years that were evaluated, as reflected by the increase in *π*_*k*_ values in both urban and rural areas. On the positive side, a significant decrease in *π*_*k*_ was found for the equity of outpatient GHS in rural healthcare facilities.

Such variations in the benefits attributed to GHS could stem from various factors, such as health resource allocation mechanisms to care-seeking behaviors that result from inappropriate health insurance schemes.

The burden of OOP expenditures may be a major contributor to the inequity in GHS benefit distribution. High user fees decrease healthcare utilization, and the poor who need medical services but cannot afford them will be excluded from the benefits [34]. We found that OOP expenditures for outpatient care decreased from 2002 to 2007 in both urban and rural areas, while inpatient expenditures increased in urban areas and remained almost unchanged in rural areas.

A decrease in OOP expenditures would be expected to improve the utilization of healthcare services, and, therefore, increase the likelihood of receiving GHS, especially by poor populations and groups needing healthcare [35, 36]. What caused the fall in OOP expenditures? Decreasing OOP expenditures for outpatients may explain why equity in government spending differed between outpatient and inpatient care, but what caused the urban–rural difference in outpatient care? In other words, since OOP expenditures for outpatient care decreased in both urban and rural areas, why did equity for ambulatory GHS improve only in rural areas? The results of this study indicate that the reasons for this apparent discrepancy may be related to China’s government healthcare resource allocation or to the coverage and benefit package of public health insurance.

Inappropriate government healthcare resource allocation may lead to higher healthcare costs, and consequently decreases economic access to medical care, especially among the poor [37]. We found that the distribution of GHS varied in different areas and among different providers. As shown in Table 4, in urban regions of Gansu province, the main GHS allocations were targeted to general hospitals, which poor patients generally do not use. On the other hand, primary healthcare facilities, which are most frequently visited by the poor, received small allocations. However, the GHS to general hospitals and primary healthcare facilities did not change over the period from 2002 to 2007. In rural areas, the allocation of subsidies was balanced across all levels of healthcare facilities. Over this period, GHS favored rural primary healthcare facilities. Government allocation of significant shares of their healthcare appropriations to low-level hospitals widens access to medical care and improves healthcare utilization among rural patients.

**Table 4:** Government health subsidy allocations in Gansu province.

		2002		**2007**	
		**Subsidy**	**Percentage**	**Subsidy**	**Percentage**
Urban	General hospital	15295.87	28.41%	48103.53	35.71%
	Traditional hospital	2076.84	3.86%	6848.26	5.08%
	Primary healthcare facility	1026.72	1.91%	1964.96	1.46%
Rural	General hospital	15559.08	28.89%	27020.55	20.06%
	Traditional hospital	4734.79	8.79%	8412.98	6.25%
	Primary healthcare facility	15155.17	28.14%	42347.04	31.44%

Data source: Author’s calculations from the local health financial yearbook.

The divergence of equity between urban and rural outpatient care might also be attributed to insurance restructuring efforts to improve coverage in rural communities and movement toward universal coverage—a policy called “Health for All”—which is taken seriously by Chinese policymakers. The expansion of rural healthcare insurance to near-universal coverage may benefit low-income groups. Table 1 also shows that the ratios of care-seeking behaviors among low-income groups in rural regions before and after NRCMS implementation changed significantly between 2002 and 2007. In 2002, about 58.33% of the poorest and 68.14% of the less poor who reported illness sought treatment. In 2007, the corresponding percentages were 71.43% and 84.00%. In urban areas, the ratio of care-seekers basically remained unchanged over this period. The increasingly frequent use of health services by the poor socioeconomic quintiles is attributed to expanded NRCMS coverage and to the allocation of government health resources among rural primary healthcare facilities—which might be key factors in improving GHS distribution equity.

The new rural healthcare insurance has focused on ensuring the breadth of universal coverage. However, other key components of universal coverage have been ignored, such as the depth and height of health insurance. A World Health Organization (WHO) study of the NRCMS reported that the total amount of pooled funds covered only 20–30% of healthcare expenditures per capita [38]. NRCMS funding is administered and implemented at the county level, but county officials are faced with choices of updating the benefits package or expanding insurance coverage. Based on the policy priorities issued by the central government, the NRCMS was designed for comprehensive population coverage at the expense of appropriate financial protections. Although the deductibles were low and reimbursable for most NRCMS enrollees, the copayment rate and ceiling were not high [39]. Diseases that could be treated with low expenditures were largely compensated by the NRCMS, but catastrophic illnesses that required hospitalization were not covered because of underfunding. It was previously reported that only 6% of hospitalization expenses were reimbursed in rural China in 2004 [40]. Therefore, care-seeking behaviors and hospital-related choices were strongly affected by the insurance scheme rather than by healthcare needs [39]. The reimbursement policies of the NRCMS were implemented to encourage many individuals, even those who needed hospitalization, to seek outpatient care rather than inpatient services. For example, many rural residents were treated with traditional Chinese medicines that were prescribed at outpatient services and accounted for up to 40% of all healthcare services delivered in China [38].

This study does have some limitations that must be acknowledged. First, we caution against generalizing our findings when debating GHS equity in China. The data were collected from a single province in China and so may not fully represent the characteristics of national healthcare benefits. Nevertheless, percentages and indices were used to evaluate how the national policies and programs were implemented in the entire population. Thus, our results are less associated with the provincial economic level and geographic location. A regional benefit equity assessment could reflect general GHS distribution to some extent. We expect that cross-province comparisons of GHS distribution equity will be performed in the future. Second, although regional economic development was considered in this study by incorporating the Gini coefficient and Kakwani index, we cannot conclude that the observed changes in GHS distribution were caused by health policy interventions, such as governmental healthcare resource allocations or NRCMS insurance. Other uncontrollable factors could affect the benefit incidence of GHS, such as differences in geographic access to healthcare, quality of health technology, and patient satisfaction. This issue should be addressed in future studies that include other socioeconomically similar provinces that do not have similar healthcare policy interventions as controls. Finally, undertaking BIA depends on the availability of data [41]. Information on GHS by type of health service was not available. We therefore made the assumption that the proportion of subsidies allocated to outpatient and inpatient care could be estimated from the ratio of outpatient and inpatient care delivered. We also assumed that all outpatient care and inpatient services (cardiac, maternity, and orthopedics in the case of inpatient care; similarly for outpatient care) were equally subsidized, respectively. It would be of value to develop more refined methods to calculate the proportion of GHS by type of health service use, in order to more accurately assess unit subsidy for different health services.

## Conclusions

The current study presents a picture of unequal distribution of GHS. Except for rural outpatient care in 2007, greater subsidies were concentrated on various healthcare initiatives among the rich and did not demonstrate inequality-reducing effects in different regions over the years studied. On the other hand, decreasing OOP expenditures and the rising allocation of government healthcare resources to primary healthcare facilities widened access and improved the opportunity to receive benefits. On the other hand, although China’s “Health for All” health insurance program improved the use and distribution of rural ambulatory care among socioeconomic groups, it drove some patients who needed hospitalization services toward outpatient visits and worsened the benefit equity of high-level hospitalized care. The trade-offs revealed here lie between providing health insurance and the proportion of the population that is covered, the range of available services, and the proportion of the total costs to be met during the progression towards universal healthcare coverage
